# Simulation of Cellular Energy Restriction in Quiescence (ERiQ)—A Theoretical Model for Aging

**DOI:** 10.3390/biology6040044

**Published:** 2017-12-12

**Authors:** David Alfego, Andres Kriete

**Affiliations:** School of Biomedical Engineering, Science and Health Systems, Drexel University, Bossone Research Center, 3141 Chestnut Street, Philadelphia, PA 19104, USA; dja58@drexel.edu

**Keywords:** retrograde response, energy stress, signaling pathways, mitochondrial dysfunction

## Abstract

Cellular responses to energy stress involve activation of pro-survival signaling nodes, compensation in regulatory pathways and adaptations in organelle function. Specifically, energy restriction in quiescent cells (ERiQ) through energetic perturbations causes adaptive changes in response to reduced ATP, NAD+ and NADP levels in a regulatory network spanned by AKT, NF-κB, p53 and mTOR. Based on the experimental ERiQ platform, we have constructed a minimalistic theoretical model consisting of feedback motifs that enable investigation of stress-signaling pathways. The computer simulations reveal responses to acute energetic perturbations, promoting cellular survival and recovery to homeostasis. We speculated that the very same stress mechanisms are activated during aging in post-mitotic cells. To test this hypothesis, we modified the model to be deficient in protein damage clearance and demonstrate the formation of energy stress. Contrasting the network’s pro-survival role in acute energetic challenges, conflicting responses in aging disrupt mitochondrial maintenance and contribute to a lockstep progression of decline when chronically activated. The model was analyzed by a local sensitivity analysis with respect to lifespan and makes predictions consistent with inhibitory and gain-of-function experiments in aging.

## 1. Introduction

The development of computational models predicting cellular adaptations in response to endogenous and external alterations has been of great interest for the biology of aging. Given the complexities of interacting mechanisms in aging, models have placed emphasis on specific aspects of the process in a descriptive manner, such as mitochondrial dynamics [1,2], protein homeostasis [3] and telomere shortening [4,5,6], as well as changes in protein connectivity with age [7,8,9]. A comprehensive integration of sub-models for mitochondria, aberrant proteins, free radicals and scavengers (MARS) has pointed a way to integrate diverse mechanisms [10]. In these implementations, Ordinary Differential Equations (ODEs) are the preferred way to compute changes in the state variables participating in the network, but it has also been demonstrated that fuzzy logic, rule-based approaches can be successful in modeling cellular aging [11]. 

An underlying question for the simulation of aging is the choice of experimental platform on which a computational model should be based upon. For instance, replicative senescence has been the most common experimental platform for examining cellular aging, but quiescence is the prevalent state in most cells and tissues [12], while brain and muscle age without involvement of replicative senescence [13]. Therefore, we focus our analysis on energy restriction in quiescence (ERiQ), which is an experimental platform for non-cycling primary cells (such as skin fibroblasts) to study responses to energetic stress induced by mitochondrial dysfunction and a simultaneous inhibition of glucose transport [14]. 

The effect of mitochondrial dysfunction on stress-signaling pathways is known as the retrograde response, which has been initially examined in yeast where mitochondrial DNA mutations happen more frequently than in vertebrates [15,16,17]. In yeast, the crosstalk between the nucleus and mitochondria adjusts metabolic function and increases longevity [18]. It has been suggested that the activation of the cell stress response factor Nuclear factor kappa B (NF-κB) in mammalian cells resembles the function of the yeast RTG gene involving alterations in metabolism and resistance to apoptosis [19,20,21,22,23]. NF-κB plays a crucial role in up-regulating inflammatory signals at the transcriptional level [24], but is activated in response to a variety of stressors [25]. In ERiQ, NF-κB activity increased with the degree of energetic perturbation. This experimental model also revealed additional factors involved in retrograde signaling, including increased protein kinase B (AKT) and suppressed tumor protein p53 expression [14].

The network model described here was initially designed as an aid to study intra-cellular signaling in response to acute energetic perturbations in quiescent cells, integrating our own experimental findings of the ERiQ phenotype with published data. The model implements elementary feedback motifs ubiquitous in engineering and places emphasis on the regulatory structure provided by key signaling nodes, implemented as a set of ODEs and functions. One core motif represents mitochondrial activity; other feedback motifs include glycolysis, p53 and the mammalian target of rapamycin (mTOR). Alterations in mitochondrial function activate connected stress sensors and signaling nodes, such as phosphatase and tensin homolog (PTEN), AKT and NF-κB, providing metabolic adaptations and cell survival. 

In the absence of precise knowledge regarding the interplay of multiple activators or inhibitors on key nodes, the model remains minimalistic and descriptive, but makes predictions consistent with the experimental platform. We hypothesized that some of the mechanisms observed experimentally and indicated computationally might occur during the aging process. The model was therefore modified by adding protein damage accumulation, leading to progressive mitochondrial dysfunction. Here we show that simulations predict stress responses observed in aging but also reveal conflicting regulatory mechanisms contributing to cellular decline. 

## 2. Materials and Methods

### 2.1. Graphical Network

**Figure 1:**
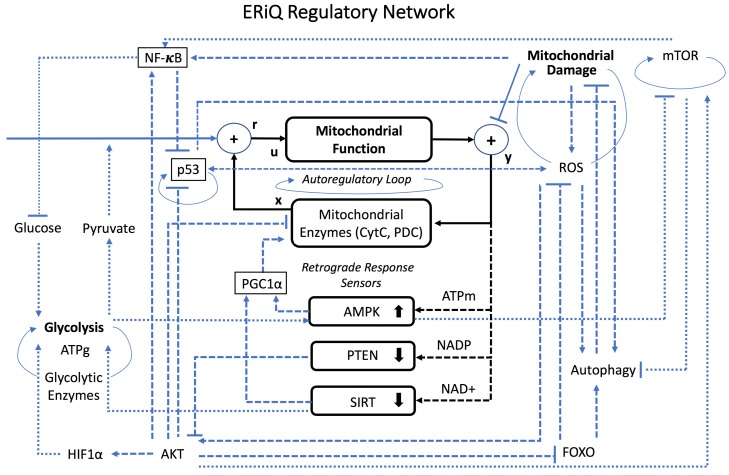
**Modular Schema of the Stress Response Network.** Integration of findings from the experimental Energy Restriction in Quiescence (ERiQ) model with knowledge of stress response signaling constitutes a comprehensive network. Autoregulatory loops (elliptic connectors) provide model stability and reject sudden perturbations. Substantial changes in the energetic state of the cell, such as a slow buildup of mitochondrial dysfunction, alter the activity of key signaling nodes involving energy stress sensors AMPK, PTEN and Sirtuins, responding to alterations in ATP, NADP and NAD+, respectively (dashed lines). The overall effect is a pro-survival phenotype comprising activation of the AKT pathway and transcription factor NF-κB, suppression of p53, and compensation of ATP production using glycolysis (dotted lines).

Prior to the program implementation, we designed a graphical schema summarizing all relevant nodes and connectivity of the model (Figure 1). The core motif is a minimalistic representation of mitochondria implemented as an autoregulatory loop. Autoregulation is a property of organelles’ capability of adjusting function to short-term fluctuations [26,27], and this negative feedback contributes to the overall stability of the model. Of note, feedbacks can provide either inhibition or activation, depending on the sign of perturbation. The level of state variables such as mitochondrial function, denoted (u) in the feedback motif, is defined by one or multiple inputs (r) and feedback (x). Inputs for mitochondria include pyruvate, p53 and Sirtuins [28,29,30,31], as well as feedback from mitochondrial enzymes (such as cytochrome C and the pyruvate dehydrogenase complex (PDC), not explicitly represented) [32]. Perturbations influence output (y), which is also the input for the negative feedback. Within the autoregulatory motifs, y tracks r and the rejection of perturbations is controlled by a gain setting. Mitochondria have been assigned a lower gain, to reflect a slower response time as compared to glycolysis. Motifs reject sudden changes through autoregulatory feedbacks, but slow and long-lasting alterations spread into the network. For instance, the first arm of the retrograde response, due to a deregulation of mitochondrial function and a decrease in oxidative phosphorylation and lower ATP, activates AMP-activated protein kinase (AMPK), which feeds back to mitochondrial function [33]. AMPK has been shown as a major regulator in mitochondrial gene expression at both basal levels and during oxidative stress. Here, activated AMPK leads to increased levels of peroxisome proliferator-activated receptor gamma coactivator-1α (PGC1a) expression, which is necessary as a regulator for metabolism in the mitochondria [34,35]. Phosphorylation of PGC1a by AMPK has even been shown to increase transcriptional activity of PGC1a, boosting mitochondrial function [35]. Increased AMPK blocks the mTOR pathway responsible for protein synthesis [36], in addition to Forkhead box (FOXO) activity, and both improve protein degradation through autophagy [37,38].

The second arm of the retrograde response involves the PI3K/AKT pathway (Figure 1), important for cell survival and proliferation, glucose metabolism and transcription. Mitochondrial dysfunction changes the NADP/NADPH ratio, which represses PTEN and its role as a negative PI3K regulator [39,40], subsequently activating AKT [41,42]. AKT leads to degradation of IKK inhibitors, activating NF-κB through enriched NF-κB-associated transcription factors, and NF-κB’s activation by AKT is controlled by mTOR [43]. AKT also binds Hypoxia-Induced Factor-1-alpha (HIF1a) [44], typically activated in low oxygen situations, with a role in glycolysis regulation [45]. AKT and NF-κB provide pro-survival mechanisms, supported by FOXO [46].

Another result of AKT activation is the inhibition of p53, which is one mechanism linking p53 suppression to mitochondrial dysfunction [47,48]. P53 is antagonistic with NF-κB, and when one pathway is activated the other is suppressed, causing distinct regulatory patterns [49,50]. Since p53 has a critical role in oxidative phosphorylation regulation, it causes dysfunction in the mitochondria. However, an overexpressed AKT can save the cell from apoptosis. Stable expression of either AKT or Bcl-2 inhibits apoptosis, but only Bcl-2 prevents the release of Cytochrome c from the mitochondria, suggesting that AKT regulates apoptosis at a mitochondrial level [51].

The third arm in the response to mitochondrial dysfunction (Figure 1) are Sirtuins [52], which are repressed when NAD+ declines [53]. Here we considered the function of SIRT1, which is a co-activator of PGC1a [54], thereby connecting to the first branch of the retrograde response. Additionally, suppression of SIRT1 contributes to mitochondrial dysfunction involving PGC1a, and activation of glycolysis. The basic setup for the glycolytic motif is similar to the mitochondrial motif. The motif has a primary regulatory feedback for autoregulation. HIF1a and Glucose are inputs, also controlled by Sirtuins [55,56]. The rate of glycolysis is increased in the network as a compensatory mechanism, but glucose uptake is progressively repressed by NF-κB, inhibiting insulin signal transduction [57].

In the absence of growth factors during quiescence, AKT levels are typically low. However, energy stress mediated by PTEN activates AKT and mTOR, specifically complex I (mTORC1). In compensation, activation of AMPK inhibits mTORC1. Increase of mTOR suppresses autophagy, as does repressed p53 [58,59]. For the aging model, we introduced an additional mitochondrial damage mechanism and the damage is dependent on and inhibited by autophagy, p53 and FOXO detoxification [60,61,62]. This module is connected to almost every branch of the model through the presence of free radicals and reactive oxygen species (ROS). ROS, normal byproducts of metabolism, typically rise in times of stress and are regulated by p53 [63]. As a result, ROS has been proven to induce autophagy [64], elevate AMPK and promote FOXO while activating AKT downstream [65]. Its presence also has stimulatory effects on NF-κB [66].

### 2.2. Computer Implementation

Implementation of the retrograde response network in a computational format required the conversion of the pathways and network nodes into a series of ODEs and functions. Feedback motifs of the model were first tested to provide homeostasis before integrating them into the larger network. The model underwent a rapid prototyping in Berkeley Madonna (v9.0, 2014, www.berkeleymadonna.com, University of California, Berkeley, CA, USA) and was subsequently implemented in Matlab (v9.2, R2017a, MathWorks, Natick, MA, USA) to solve a total of seven ODEs involved in mitochondrial function (mFUNCT), Glycolysis (GLYCOL), p53 and mitochondrial damage (mDAMAGE) (see File S1 for the Matlab code, using the stiff solver ODE15s).

Following an earlier approach [11], inputs (u) for the state variables responsible for mitochondrial function and ATP output (ATPm) and glycolytic function and ATP output (ATPg) were adjusted to reflect a 60:40 ratio, as shown by experimental findings [67]. Response rates (g) in these feedback motifs were adjusted, i.e., a fast response was chosen for the glycolysis motif and a slow response for the mitochondrial motif. Subsequently, feedback motifs were connected to signaling nodes and auxiliary variables, and these activating or inhibiting interactions are provided in Table S1, along with references. Energetic stress sensors PTEN and SIRT1 correlate with mitochondrial function, and AMPK correlates inversely with intracellular ATP. The interaction strength of main signaling nodes was estimated from the experimental platform with respect to energetic perturbations, as provided by Western blots or assays. The experiments provided changes of the state variables NF-κB, AKT and mTOR with respect to low ATP levels [14]. In cases of multiple interactions, we assumed linear combinations of all interacting species. For instance, p53 in our model is equally inhibited by NF-κB and AKT.

The unperturbed model approached steady-state levels and initial values of state variables were adjusted accordingly to support convergence. The homeostatic condition was used to examine recovery from short, pulsed perturbations. When mitochondrial damage rates (MDR) were introduced and set to values >1.5 × 10^−3^, the system did not recover and mitochondrial function started to decline, simulating aging. The MDR rate of 1.8 × 10^−3^ was chosen to establish a model runtime of approximately 800. However, the overall dynamic of the model did not change substantially when higher or lower damage rates were applied. The model lifetime ended when mitochondrial energy (ATPm) output dropped below 0.5 (from an initial vale of 3.5).

It is clear this is only the first step towards a more detailed model reflecting measurable rates as they become available. 

### 2.3. Model Analysis

Due to the complicated nature of the network, it is necessary to test the behavior of many of its components to determine how the overall system depends on them [68]. To test how the system recovers from specific perturbations during its runtime, a “pulsed” event was placed in the timeline to disrupt normal conditions. We performed a series of local sensitivity analyses, which are invaluable to understanding the relevance of key system parameters [69]. The first analysis perturbed each of the major components of the motifs by a consistent 10% increase and compared the results to those of normal conditions. A sensitivity objective function (SOF) [69] was then calculated as a ratio to compare how these perturbations affect both total duration of the system and their impact on mitochondrial function (mFUNCT) at a time-point always reached amongst all perturbations (t = 500). SOF was calculated as the percent change in mFUNCT or lifespan divided by the percent change in rate (always 10%). Parameters tested were: PTEN, AKT, NF-κB, p53, AMPK, PGC1a, mTOR, autophagy, FOXO, HIF1a and SIRT.

A second analysis measured the duration of the model’s lifetime through a combination of increasing and decreasing relative p53 and NF-κB activity versus different relative mitochondrial damage rates. P53 was increased via a p53 activator (p53_Act) in a range of 0.4 to 4.0. Mitochondrial damage rate (MDR) was varied within the range of 1.5 × 10^−3^ to 2.6 × 10^−3^.

## 3. Results

### 3.1. Simulations and Perturbations

**Figure 2:**
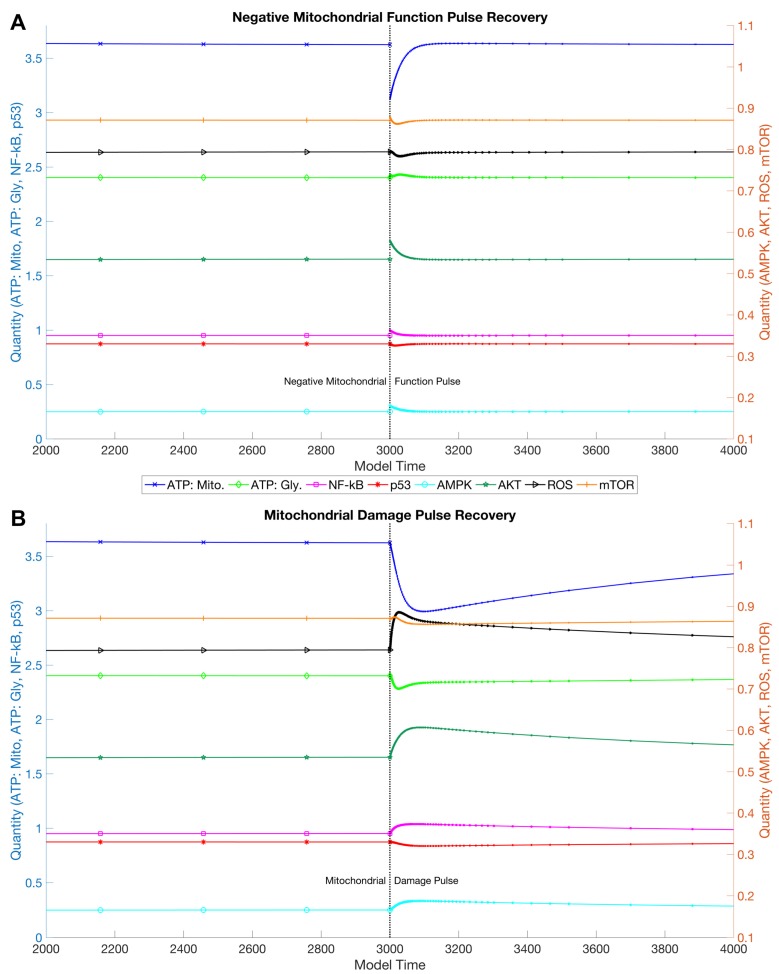
**Simulation of System Recovery after Energetic Perturbation**. The computation shows the reactions of selected network components running at homeostatic conditions when introduced to sudden changes in the system. Both panels are double axes plots with ATP production, NF-κB and p53 on the left axis (blue) and AMPK, ROS, AKT and mTOR activity on the right axis (red); both share the same legend. (**A**) A “negatively pulsed” decrease in mitochondrial function results in immediate ATP production loss due to compromised oxidative phosphorylation. Increases in AMPK, NF-κB activity and ATP production from glycolysis recover the system. (**B**) A “pulsed” increase in mitochondrial damage is accompanied by a steep decrease in mitochondrial ATP production. There is a noticeable increase in NF-κB and AMPK activity, as well as ROS levels, while p53 decreases. The system required a longer period to recover due to limited damage removal.

The model’s primary goal was to chart mitochondrial function in response to acute perturbations, followed by a lifespan simulation. A sudden decrease in mitochondrial function (mFUNCT) under homeostatic conditions caused a decrease in ATP produced by the mitochondria (Figure 2, Panel A). The system adapted by increasing the rate of glycolysis to compensate. The event also induced activation of AMPK and NF-κB, and a drop in p53. This would be equivalent to adding a mitochondrial uncoupler to a cell culture, and removing it after a period of time. The results are therefore consistent with the findings of the experimental ERiQ platform, where inhibitors were applied to quiescent fibroblasts for 24 h, after which they were removed and cells recovered. Increase of mitochondrial dysfunction and limitations in glucose uptake further increased energy stress and NF-κB activity [14]. 

Mitochondrial dysfunction was alternatively introduced by a step increase in mitochondrial damage (mDAMAGE) (Figure 2, Panel B). This affected mitochondrial function, increased ROS levels and decreased mitochondrial ATP production. Also examined were increases in NF-κB and AMPK, and diminished p53. The adaptation of stress-signaling nodes was similar to the earlier simulation; however, recovery occurred later due to slower process of damage elimination.

**Figure 3:**
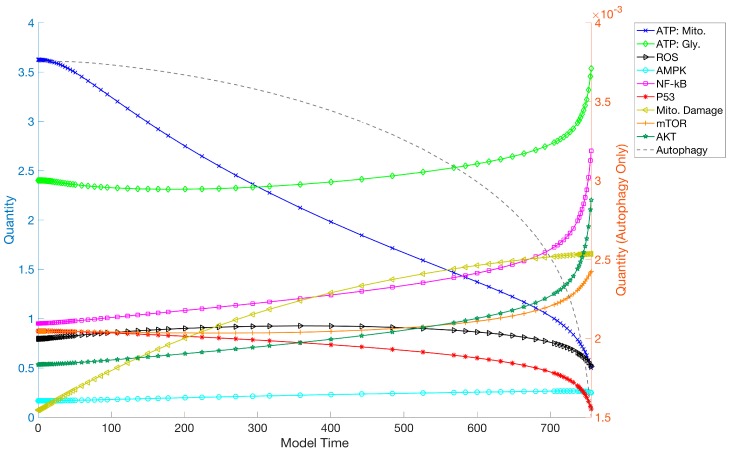
**Simulation of the Aging Model**. In this simulation of the ERiQ model, the concentration of ATP produced via mitochondria decreases due to a lack of mitochondrial maintenance. AKT and NF-κB steadily increase, inhibiting p53. The signaling nodes also cause an upregulation in glycolytic ATP production to compensate for low energy. However, total ATP levels decline slightly, upregulating AMPK. There are smaller changes in mTOR and ROS levels, in contrast to autophagy, which declines strongly (shown on its own scale). The model runs until ATPm reaches a low level, here a model time of t = 756 was recorded.

Moving the model away from steady-state values by increasing mitochondrial damage rates beyond the capabilities of system recovery allowed the simulation of aging in quiescent cells (Figure 3). The graph shows quantities of ATPm and ATPg, ROS, AMPK, NF-κB, p53, mTOR, AKT and mDAMAGE over a lifespan. NF-κB, AKT and AMPK slowly increased with time, while p53 declined and mTOR remained almost constant until approaching termination of the model. ATPg production slightly increased as a compensation for low ATPm output, yet due to the inhibitory role of NF-κB on glucose uptake, the increase was only moderate.

### 3.2. Sensitivity Analysis

**Figure 4:**
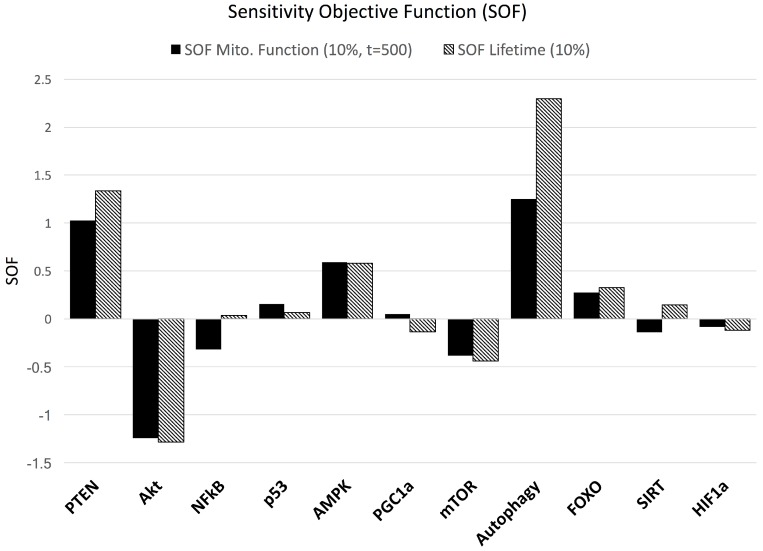
**Sensitivity Analysis of Major Network Nodes**. The local sensitivity analysis was performed by perturbing each of the major nodes of the system by +10%. Resulting Sensitivity Objective Functions (SOFs) were calculated for mitochondrial function at (t = 500), and for terminal lifespan. Perturbations in AKT, mTOR and HIF1a all resulted in decreases in both mitochondrial function and lifespan, while PTEN, p53, AMPK and FOXO caused the opposite. Increased autophagy indicates the strongest effect in extending lifespan. NF-κB, PGC1a and SIRT have opposing effects between the two measurements for the selected readout-time.

The results of perturbations of the major signaling nodes are reflected in a bar chart (Figure 4), consisting of SOF values for both lifespan and mitochondrial function. Negative changes in both lifespan and mitochondrial function (mFUNCT) with respect to a 10% increase in AKT, mTOR and HIF1a were determined. An increase in lifespan and mFUNCT occurred in the perturbations of PTEN, p53, AMPK, autophagy and FOXO. Autophagy perturbations resulted in the highest sensitivity for both model duration and mFUNCT. Changes in NF-κB and SIRT saw increases in lifetime, but a decrease in mFUNCT, while PGC1a saw the opposite for the selected timepoint. 

**Figure 5:**
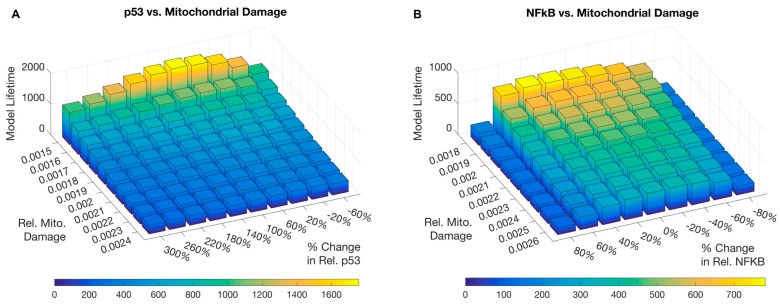
Model Runtimes for p53 and NF-κB Variants. A closer examination of the sensitivities of ranges of p53 and NF-κB with respect to a range of mitochondrial damage rates reveals optimal setting points for maximal lifespan. (**A**) Relative mitochondrial damage was compared to positive and negative changes in p53 activity. Results show bell-curve distributions that demonstrate shortened lifespan if p53 activity is too low, or too high. (**B**) Lifetimes with respect to NF-κB activity show a similar pattern. In comparison to the normal settings of the model (Figure 3), only an increase in p53 extends lifespan.

The second sensitivity analysis compared varying quantities of selected parameters over a larger range, allowing identification of optimal values with respect to lifespan. A p53 analysis is represented as a three-dimensional comparison of lifetimes for a range of mitochondrial damage rates versus a range of p53 activations (P53_Act) (Figure 5, Panel A). Activity of p53 has an optimal range shortening lifespan if too low or too high, independent of damage rates. Our second analysis shows a similar outcome between relative damage rates and NF-κB activity (Figure 5, Panel B). With respect to the default settings of the model, p53 is too low to reach maximal lifespan, and NF-κB is insensitive over a range of variations.

## 4. Discussion

The current model’s primary purpose was to integrate experimental findings with current knowledge on cellular stress responses to make system predictions with respect to acute and chronic energetic perturbations. The model is theoretical and descriptive due to the lack of suitable data, which has been identified as the main hindrance for more comprehensive cell modeling endeavors [70]. However, development of computer representations and experimentation in systems biology are always part of an iterative process and the model described here can serve as a catalyst to direct future experimental work, which in return will advance model detail. Specifically, we did not consider enzyme kinetics, since we assumed that reactions do not change with age and the system remains in homeostasis on shorter timescales. In addition, we had to simplify many interactions and components, such as Sirtuins and FOXO proteins, which, due to lack of exact knowledge of their interrelationships and quantitative effect sizes, could not be modeled in more depth. We also did not include transport mechanisms in and out of mitochondria and the nucleus. Nevertheless, such data can be added when it becomes available, and the experimental platform ERiQ provides an opportunity to further identify and quantify parameters that may be of relevance in aging.

The model demonstrates pro-survival mechanisms in response to a pulse of mitochondrial dysfunction, avoiding cell death through elevated AKT and NF-κB, but reduced p53 levels, allowing the cell to recover when energetic stress is removed (Figure 2). However, these mechanisms seem to be in conflict with long-term cellular performance (Figure 3). A prime example is the activation of AKT in the progression of aging, suppressing p53 required for mitochondrial function and with a role in autophagy. This activates the mitochondrial inhibitor HIF1a, which conflicts with PGC1a activated by AMPK. The observation of an active downregulation of a subset of mitochondrial genes coded in the nuclear DNA [71,72] is therefore consistent with the model. Inhibition of AKT improves lifespan (Figure 4), which has been shown experimentally in vitro and in vivo [73,74]. Furthermore, our simulation outcomes corroborate observations of a shift in metabolism from oxidative phosphorylation to glycolysis, involving Sirtuins, also described as a “pseudohypoxic” state [75]. 

The activity of mTOR remains relatively unchanged over the model’s lifetime, as demonstrated in mice [76], due to the opposing roles of AKT and AMPK. The constitutive activation of the pro-survival NF-κB protein complex by a cell-intrinsic, “atypical” mechanism [77], which inhibits glucose uptake when overexpressed, likely contributes to epigenetic and pathophysiological changes not reflected in the model. Not only does it change the epigenetic dynamics [78], but chronic inflammation is generally considered a risk factor in age-associated diseases like cancer, arthritis and cardiovascular disease, and genetic variants within the NF-κB network have been associated with longevity [79]. Consequently, modulation of NF-κB is considered a strategy to influence aging [80] and both hyperactivity of NF-κB [81], as well as its loss of function [82,83], reduced lifespan. The model indicates that ROS levels do not have to increase for the accumulation of permanent damage, as demonstrated earlier [11].

Similarly, repression of p53, which competes in an antagonistic fashion with NF-κB over a common pool of transcriptional co-activators [50], serves an anti-apoptotic role in acute phases of energy stress. However, the p53 pathway is engaged in DNA repair mechanisms like base excision repair, which also includes mtDNA repair [84]. Only if p53 is activated by stronger signals, like telomere shortening in proliferating cells [85], the situation changes—cells enter apoptosis or enter a senescent state [86]. It would be of interest to validate the predicted decline in activity of p53 in post-mitotic tissues in vivo, since p53 is upregulated in senescence, but declines with age in the population [87]. In our model, an optimal level for p53 can be found maximizing lifespan (Figure 5). Activation of p53 initially increases lifespan due to its involvement in mitochondrial function, but stronger activation shortens it. This finding may reflect in part more complex mechanisms studied in p53 mutant mice [88]. 

## 5. Conclusions

In summary, predictions of the computational ERiQ model show consistency with experimental findings in post-mitotic cells and aging models. The stress-signaling network identified here extends beyond components of the retrograde response to previously less discussed signaling nodes of interest for the systems biology of aging [89,90,91], providing an entry point for an in-depth multiscale modeling of metabolic regulation. Future work may further delineate conflicting cellular control mechanisms, which are an example of a robustness tradeoff in evolutionary design [92]. Evolved responses provide short-term pro-survival advantages, but the very same mechanisms contribute to a lockstep decline and deleterious effects of aging.

## Supplementary Materials

The following are available online at www.mdpi.com/2079-7737/6/4/44/s1, File S1: Matlab code, Table S1: State variable interactions.
