# Supplementary material for: Simulation of Cellular Energy Restriction in Quiescence (ERiQ)—A Theoretical Model for Aging
Source: Biology (Basel). 2017 Dec 12;6(4):44. doi: 10.3390/biology6040044 (PMC5745449; doi:10.3390/biology6040044)
Supplement: Supplementary file 1 [file biology-06-00044-s001.pdf]

File S1 - Energy Restriction in Quiescence Simulation Code

Network of energy restriction as a model of aging using Matlab®. Code published in the following order: ERiQ model, node functions in alphabetical oder, ODE solver and plotting.

Authors: David Alfego (1), Andres Kriete (1)

(1) School of Biomedical Engineering, Science and Health Systems, Drexel University, Philadelphia, PA

Contents

- Establish ERiQ model
- Akt Node
- AMPK Node
- ATP Node
- Autophagy Node
- FOXO Node
- Free Radical Node
- Glucose Node
- Glycolysis ODE
- Glycolytic Enzymes ODE
- HIF Node
- Mitochondrial Damage Rate Node
- Mitochondrial Damage ODE
- Mitochondrial Enzymes ODE
- Mitochondrial Function ODE
- mTOR Node
- mTOR Regulatory Feedback ODEs
- NAD Node
- NFkB Node
- p53 Node
- p53 Regulatory Feedback ODEs
- PGC1alpha Node
- PTEN Node
- Pyruvate Node
- ROS Node
- ROS Regulatory Feedback ODEs
- Sirtuin Node
- Event to stop simulation
- ODE Solving

Establish ERiQ model

Created equations based on graphical model

```
function dY = ERiQ(t,Y)

%Name variables
MDAMAGE = Y(1);
MFUNCT = Y(2);
MENZY = Y(3);
GLYCOL = Y(4);
GLYENZ = Y(5);
Cy = Y(6); Ay = Y(7);
Cx = Y(8); Ax = Y(9);
Cz = Y(10); Az = Y(11);

[ATPm,ATPg,ATPr] = f_ATP(GLYCOL, MFUNCT);

% ROS Module
ROS = f_ROS(Az);
% {* x, at > 0.5 runtime > 900, flips at > 5.0 to reduced runtimes}
PTEN = f_PTEN(MFUNCT);
AKT = f_AKT(PTEN,ROS);

% mTOR
AMPK = f_AMPK(ATPr);
NADr = f_NADr(MFUNCT);
% { NAD+/NADH ratio}
SIRT = f_SIRT(NADr);
PGC1alpha = f_PGC1a(AMPK,SIRT);
[MTORs,MTORa,MTOR] = f_MTOR(AKT,AMPK,Ay);
NFKB = f_NFKB(AKT,ROS,MTOR);
% { negative feedback }

% p53 Autoregulation
[P53s,P53a,P53] = f_P53(AKT,NFKB,ROS,Ax);

% ROS cont.
FOXO = f_FOXO(AKT);
% { FOXO detoxifies, is inhibited by AKT }
Uz = f_FREERAD(P53,MDAMAGE,Cz,FOXO);
% Free Radicals

% Autophagy Module
AUTOPHAGY = f_AUTO(MTOR,FOXO,ROS,P53);
% { Autophagy depends on mTOR and P53 }

% Mito / Apoptosis
HIF = f_HIF(AKT);
PYR = f_PYR(GLYCOL);
% { a function of Glycolysis }
r2 = (PGC1alpha+PYR+P53)-(HIF*0.2)-(NFKB*0.2);
% { positive feedback }
MD = f_MD(MFUNCT,ROS);
% { this rate will be modified slightly up and down }
% { <<<<<< constant scales damage accumulation in next equation, range }
d2 = MDAMAGE;
k4 = (r2-d2);
gain2 = 0.05;
% { >>> gain determines speed of response, mitochondria respond
%slower than glycolysis }
u2 = r2+MENZY;

% Glycolysis
```

```
GLU = f_GLU(NFKB);
% { GLU is Glucose uptake, inhibited by NFkB, should remain positive }
r3 = GLU+(HIF*.01)+(NADr*.01);
u3 = r3+GLYENZ;
k6 = r3;

% ODEs
dY = zeros(11,1);
dY(1) = f_MDAMAGE(MD,AUTOPHAGY);                % Mitochondrial Damage
dY(2) = f_MFUNCT(gain2,u2,SIRT);                % Mitochondrial Function
dY(3) = f_MENZY(ATPm,k4,MENZY);                 % Mitochondrial Enzymes
dY(4) = f_GLYCOL(u3,SIRT);                      % Glycolysis
dY(5) = f_GLYENZ(ATPg,k6,GLYENZ);               % Glycolytic Enzymes
[dY(6),dY(7)] = f_MTOR_feedback(MTORa,Cy);      % mTOR Feedback loop
[dY(8),dY(9)] = f_P53_feedback(P53a,Cx);        % p53 Feedback loop
[dY(10),dY(11)] = f_ROS_feedback(ROS,Cz,Uz);    % ROS Feedback loop

end
```

Akt Node

Akt equation dependent on PTEN inhibition and Reactive Oxygen Species (ROS) activation.

```
function AKT = f_AKT(PTEN,ROS)

global AKT_SA

GF = 0.1;
AKT = AKT_SA*(GF+PTEN+(ROS/5));

end
```

AMPK Node

AMPK equation dependent on activation by total ATP (ATPr) produced).

```
function AMPK = f_AMPK(ATPr)

global AMPK_SA

AMPK = AMPK_SA*(1./ATPr);

end
```

ATP Node

ATP produced by the mitochondria (ATPm) dependent on mitochondrial function (MFUNCT). ATP produced via glycolysis (ATPg) dependent on glycolysis equation (GLYCOL). Total ATP = ATPr.

```
function [ATPm,ATPg,ATPr] = f_ATP(GLYCOL, MFUNCT)

ATPg = GLYCOL;
ATPm = MFUNCT;
ATPr = ATPg+ATPm;

end
```

Autophagy Node

Autophagy equation dependent on activation by mTOR, FOXO, p53 and Reactive Oxygen Species (ROS).

```
function AUTOPHAGY = f_AUTO(MTOR,FOXO,ROS,P53)

global AUTO_SA
Scale1 = 0.001;

AUTOPHAGY = AUTO_SA*(Scale1*((1./MTOR)+(FOXO*0.5)+ROS+P53));

end
```

FOXO Node

FOXO equation dependent on AKT activation.

```
function FOXO = f_FOXO(AKT)

global FOXO_SA

FOXO = FOXO_SA*(1./AKT);

end
```

Free Radical Node

Free Radical (FREERAD) equation dependent on activation by p53 and mitochondrial damage (MDAMAGE) and inhibition by FOXO.

```
function Uz = f_FREERAD(P53,MDAMAGE,Cz,FOXO)

global FREERAD_SA

Uz = FREERAD_SA*(P53+(MDAMAGE*0.2)+Cz-(FOXO*.05));

end
```

Glucose Node

Glucose quantity equation dependent on NFkB activation.

```
function GLU = f_GLU(NFKB)

global GLU_SA

GLU = GLU_SA*(1./(NFKB));

end
```

Glycolysis ODE

Glycolysis (GLYCOL) equation dependent on activation by Sirtuins (SIRT) and established u3 from main function ERiQ.m.

```
function GLYCOL = f_GLYCOL(u3,SIRT)

global GLYCOL_SA

gain3 = 0.25;

GLYCOL = GLYCOL_SA*((gain3*u3)+(1./SIRT));

end
```

Glycolytic Enzymes ODE

Glycolytic Enzymes (GLYENZ) equation dependent on ATP produced from glycolysis activation and inhibition from k6 established in main function ERiQ.m and current enzyme quantity.

```
function GLYENZ = f_GLYENZ(ATPg,k6,GLYENZ)

k5 = -1;

GLYENZ = (k5*ATPg)-(k6*GLYENZ);

end
```

HIF Node

HIF equation dependent on Akt activation.

```
function HIF = f_HIF(AKT)

global HIF_SA

HIF = HIF_SA*AKT;

end
```

Mitochondrial Damage Rate Node

Mitochondrial damage rate (MD) dependent on mitochondrial function (MFUNCT) and Reactive Oxygen Species (ROS) activation.

```
function MD = f_MD(MFUNCT,ROS)

global MDR MDR_SA
%ScaleFactor represents average ROS that will provide no change in normal
%conditions, but should ROS become elevated, the scaling factor allows for
%realistic changes outside the autoregulatory loop.
ScaleFactor = 0.8;

MD = MDR_SA*(((abs(MFUNCT+ROS))*MDR)+((ROS-ScaleFactor)*0.0001));

end
```

Mitochondrial Damage ODE

Mitochondrial damage (MDAMAGE) equation dependent on damage rate (MD) and Autophagy inhibition.

```
function MDAMAGE = f_MDAMAGE(MD,AUTOPHAGY)

global MDAMAGE_SA

MDAMAGE = MDAMAGE_SA*(MD-AUTOPHAGY);

end
```

Mitochondrial Enzymes ODE

Mitochondrial Enzymes (MENZY) equation dependent on ATP production from the mitochondria (ATPm) activation and current enzyme quantity inhibition.

```
function MENZY = f_MENZY(ATPm,k4,MENZY)

k3 = 1;

MENZY = -(k3*ATPm)-(k4*MENZY);

end
```

Mitochondrial Function ODE

Mitochondrial function (MFUNCT) equation dependent on SIRT inhibition and established u2 activation from main function.

```
function MFUNCT = f_MFUNCT(gain2,u2,SIRT)

MFUNCT = (gain2*u2)-(SIRT*.02);

end
```

mTOR Node

mTOR equation dependent on AKT activation and AMPK inhibition.

```
function [MTORs,MTORa,MTOR] = f_MTOR(AKT,AMPK,Ay)

global MTOR_SA

MTORs = (AKT-(AMPK*4));
MTORa = Ay-(MTORs*1.5);

MTOR = MTOR_SA*(1+MTORs+MTORa);

end
```

mTOR Regulatory Feedback ODEs

mTOR feedback loop equation dependent on current mTOR quantity.

```
function [Cy,Ay] = f_MTOR_feedback(MTORa,Cy)

ry = 0;
gy = 0.1;
```

```
Uy = (ry+Cy);

Cy = -MTORa-Cy;
Ay = gy*Uy;

end
```

NAD Node

NAD ration equation dependent on mitochondrial function (MFUNCT) activation.

```
function NADr = f_NADr(MFUNCT)

global NADr_SA

%NADr is ratio
NADr = NADr_SA*MFUNCT;

end
```

NFkB Node

NFkB equation dependent on Akt, mTOR and Reactive Oxygen (ROS) activation.

```
function NFKB = f_NFKB(AKT,ROS,MTOR)

global NFKB_SA

NFKB = NFKB_SA*(AKT+(ROS*0.25)+(MTOR*0.25))*1;

end
```

p53 Node

p53 equation dependent on Reactive Oxygen Species (ROS) activation and Akt, NFkB inhibition.

```
function [P53s,P53a,P53] = f_P53(AKT,NFKB,ROS,Ax)

global P53_Base P53_Act P53_SA
%P53Act = 1 in script {< 1 = no activation, modify this between 0.1 and 4};

P53s = 0.3*(P53_Base-(AKT)-(NFKB)+(ROS*0.5))*P53_Act;
P53a = Ax-P53s;
%{y, output tracks r}
P53 = P53_SA*(P53s + P53a);

end
```

p53 Regulatory Feedback ODEs

p53 feedback equation dependent on current p53 quantity.

```
function [Cx,Ax] = f_P53_feedback(P53a,Cx)
```

```
rx = 0;
% { Zero P53a output if no change }
gx = 0.1;
% { smooth close to 0 behaivior long term }
Ux = (rx+Cx);

Cx = -P53a-Cx;
Ax = gx*Ux;

end
```

PGC1alpha Node

PGC1alpha (PGC1a) equation dependent on AMPK and Sirtuin (SIRT) activation.

```
function PGC1alpha = f_PGC1a(AMPK,SIRT)

global PGC1a_SA

PGC1alpha = PGC1a_SA*(AMPK+(SIRT*0.1));

end
```

PTEN Node

PTEN equation dependent on mitochondrial function (MFUNCT) and its activity is inversely implemented.

```
function PTEN = f_PTEN(MFUNCT)

global PTEN_SA

PTEN = PTEN_SA*(1./MFUNCT);

end
```

Pyruvate Node

Pyruvate (PYR) equation dependent on Glycolysis (GLYCOL) activation.

```
function PYR = f_PYR(GLYCOL)

global PYR_SA

PYR = PYR_SA*GLYCOL*0.7;

end
```

ROS Node

Reactive Oxygen Species (ROS) equation dependent on regulatory feedback, Az.

```
function ROS = f_ROS(Az)

global ROS_SA
```

```
ROS = Az*10*ROS_SA;

end
```

ROS Regulatory Feedback ODEs

Reactive Oxygen Species (ROS) feedback equation dependent on current ROS quantity and established Free Radical equation, Uz.

```
function [Cz,Az] = f_ROS_feedback(ROS,Cz,Uz)

gz = .01;

Cz = -ROS-Cz;
Az = gz*Uz;
%Uz defined in ERiQ.m

end
```

Sirtuin Node

Sirtuin equation (SIRT) dependent on NAD ratio (NAD) activation.

```
function SIRT = f_SIRT(NADr)

global SIRT_SA

SIRT = SIRT_SA*NADr;

end
```

Event to stop simulation

End simulation when mitochondrial function (MFUNCT) = 0.5 for accuracy.

```
function [value,isterminal,direction] = event_retrograde(t,Y)

value = Y(2)-.5;  %Detect ATPt=0
isterminal = 1; %Stop the fcn
direction = 0;

end
```

ODE Solving

Run ode15s for stiff equations using established homeostatic initial conditions. The simulation is terminated when MFUNCT=0.5 for accuracy. Global parameters are constant that can be changed to increase or decrease nodes for Sensitivity Analysis. Remainder of script produces a plot of major nodes and a table that tracks final node concentration.

```
function ERiQ_ODE
t0 = [0,10000];

% Conditions to establish homeostais: Y0 = [0 3 1 2 0 0 0 0 1 0 0];
%   Order: [MDAMAGE MFUNCT MENZY GLYCOL GLYENZ Cy Ay Cx Ax Cz Az]
```

```
% Homeostatis Initial Conditions:
Y0 = [0.0724    3.6239   -1.3358    2.4010   -2.1968   -0.0000   ...
-0.1936   -0.0000    0.8734   -0.7944    0.0794];
%   For pulse simulation, these initial conditions can be changed ...
%   at specified time to indicate sudden increase in MDAMAGE or MFUNCT

% End simulation when MFUNCT=0.5
options = odeset('Events',@ERiQ_event);

% Modified Damage and p53 rates using global constants across functions
%   Can change to obtain values for 3d multiple parameter plots
global P53_Base P53_Act MDR
P53_Base = 4;
P53_Act = 1;
MDR = 1.8E-3;
% Ideal conditions

% Global used for constants only - for quick sensitivity analysis
%   i.e. change global constant from 1 to 1.1 for 10% increase in node
global PTEN_SA AKT_SA FREERAD_SA NFKB_SA P53_SA AMPK_SA PGC1a_SA ...
    MTOR_SA MDAMAGE_SA MDR_SA PYR_SA AUTO_SA FOXO_SA NADr_SA ...
    GLYCOL_SA GLU_SA HIF_SA SIRT_SA ROS_SA

ROS_SA = 1; PTEN_SA = 1; FREERAD_SA = 1; AKT_SA = 1; NFKB_SA = 1;
P53_SA = 1; AMPK_SA = 1; PGC1a_SA = 1; MTOR_SA = 1; MDAMAGE_SA = 1;
MDR_SA = 1; PYR_SA = 1; AUTO_SA = 1; FOXO_SA = 1; NADr_SA = 1;
GLYCOL_SA = 1; GLU_SA = 1; HIF_SA = 1; SIRT_SA = 1;

[t,Y] = ode15s(@ERiQ,t0,Y0,options);

MDAMAGE = Y(:,1);
MFUNCT = Y(:,2);
MENZY = Y(:,3);
GLYCOL = Y(:,4);
GLYENZ = Y(:,5);
Cy = Y(:,6); Ay = Y(:,7);
Cx = Y(:,8); Ax = Y(:,9);
Cz = Y(:,10); Az = Y(:,11);

% Nodes for analysis
[ATPm,ATPg,ATPr] = f_ATP(GLYCOL, MFUNCT);
ROS = f_ROS(Az);
PTEN = f_PTEN(MFUNCT);
AKT = f_AKT(PTEN,ROS);
AMPK = f_AMPK(ATPr);
PYR = f_PYR(GLYCOL);
FOXO = f_FOXO(AKT);
[MTORs,MTORa,MTOR] = f_MTOR(AKT,AMPK,Ay);
NFKB = f_NFKB(AKT,ROS,MTOR);
NADr = f_NADr(MFUNCT);
[P53s,P53a,P53] = f_P53(AKT,NFKB,ROS,Ax);
AUTOPHAGY = f_AUTO(MTOR,FOXO,ROS,P53);
MD = f_MD(MFUNCT,ROS);
GLU = f_GLU(NFKB);
HIF = f_HIF(AKT);
SIRT = f_SIRT(NADr);
PGC1alpha = f_PGC1a(AMPK,SIRT);
Uz = f_FREERAD(P53,MDAMAGE,Cz,FOXO);

duration = t(end);
```

```
% Plot Major Nodes
figure(1);
hold on
plot(t,ATPm,'b',t,ATPg,'g',t,ROS,'k',t,AMPK,'c',t,NFKB,'m',t,P53, ...
    'r','LineWidth',2);
plot(t,MDAMAGE,'Color',1/255*[200,200,0],'LineWidth',2);
plot(t,MTOR,'Color',[1 .5 0],'LineWidth',2);
plot(t,AKT,'Color',1/255*[0,150,87],'LineWidth',2);
xlabel('Time','FontSize',14);
ylabel('Quantity (mTOR, ATP: Mito, ATP: Gly, ROS, AMPK)','FontSize',14);
legend('ATP: Mito.','ATP: Gly.','ROS','AMPK','NFKB','P53', ...
    'Mito. Damage','MTOR','AKT')
hold off

% Parameter Table

% Sensitivity Analysis - node parameter perturbations, can change
% simulation ending time to evaluate MFUNCT (i.e. at t=500) or run until
% end to see how perturbation changes lifespan. Print out final values for
% each node in table

mfunctend = repelem(MFUNCT(end),18);
life = repelem(t(end),18);
X = [PTEN(end) ROS(end) AKT(end) NFKB(end) P53(end) AMPK(end) ...
    PGC1alpha(end) MTOR(end) MDAMAGE(end) MD(end) PYR(end) ...
    AUTOPHAGY(end) FOXO(end) NADr(end) GLYCOL(end) GLU(end) HIF(end) ...
    SIRT(end); mfunctend; life]';
colNames = {'Parameter','MFUNCT','Life'};
rowNames = {'PTEN','FREERAD','AKT','NFKB','P53','AMPK','PGC1a','MTOR', ...
    'MDam','MD','PYR','AUTO','FOXO','NADr','GLYCOL','GLU','HIF','SIRT'};
Parameters = array2table(X,'RowNames',rowNames,'VariableNames',colNames);

end
```

Published with MATLAB® R2016a

Table S1 – State Variable Interactions

Node	Connection	Action	Reference
**AKT**	PTEN	Inhibition	[42] Guha, M., et al.
	ROS	Activation	[65] Zhao, Y., et al.
**AMPK**	ATPr	Activation	[33] Jeon, S.M.
**AUTOPHAGY**	FOXO	Activation	[38] Sandri, M.
	MTOR	Activation	[58] Jung, C.H., et al.
	P53	Activation	[59] Maiuri, M.C., et al.
	ROS	Activation	[64] Filomeni, G.D., et al.
**FOXO**	AKT	Activation	[46] Nakae, J., et al.
**GLU**	NFKB	Inhibition	[57] de Luca, C., et al.
**Glycolysis**	GlyEnz	Activation	[45] Yeung, S.J., et al.
	SIRT	Activation	[55] Lim, J.H., et al.<br>[56] Rodgers, J.T., et al.
**GlyEnz**	Glycolysis	Activation	[45] Yeung, S.J., et al.
**HIF**	AKT	Activation	[44] Gorlach, A., et al.
**MEnzy**	MFunct	Inhibition	[32] Chen, Q., et al.
**MFunct**	MEnzy	Activation	[32] Chen, Q., et al.
	P53	Activation	[28] Matoba, S., et al.<br>[29] Donahue, R.J., et al.
	PYR	Activation	[30] Lombard, D.B., et al.
	SIRT	Inhibition	[31] Brenmoehl, J., et al.
**MDamage**	AUTOPHAGY	Inhibition	[60] Van Houten, B., et al.
**MTOR**	AKT	Activation	[37] Polak, P., et al.
	AMPK	Inhibition	[36] Gwinn, D.M., et al.
**NADr**	MFunct	Activation	[53] Ying, W.
**NFKB**	AKT	Activation	[43] Dan, H.C., et al.
	MTOR	Activation	[43] Dan, H.C., et al.
	ROS	Activation	[66] Morgan, M.J., et al.
**P53**	AKT	Inhibition	[47] Compton, S., et al.
	NFKB	Inhibition	[49] Webster, G.A., et al.
	ROS	Activation	[63] Liu, B., et al.
**PGC1alpha**	AMPK	Activation	[35] Jager, S., et al.
	SIRT	Activation	[54] Canto, C., et al.
**PTEN**	MFunct	Activation	[40] Song, M.S., et al.
**PYR**	Glycolysis	Activation	[57] de Luca, C., et al.
**ROS**	FOXO	Inhibition	[65] Zhao, Y., et al.
	MDamage	Activation	[64] Filomeni, G.D., et al.
	P53	Activation	[63] Liu, B., et al.
**SIRT**	NADr	Activation	[53] Ying, W.

Table S1. State Variable Interactions. A list of each major node in the computational model and the corresponding node that acts on it. Activity is defined as either inhibiting or activating. Matching references from the article bibliography support these connections.
